# Human bone marrow- and adipose-mesenchymal stem cells secrete exosomes enriched in distinctive miRNA and tRNA species

**DOI:** 10.1186/s13287-015-0116-z

**Published:** 2015-07-01

**Authors:** Serena Rubina Baglio, Koos Rooijers, Danijela Koppers-Lalic, Frederik J. Verweij, M Pérez Lanzón, Nicoletta Zini, Benno Naaijkens, Francesca Perut, Hans W. M. Niessen, Nicola Baldini, D. Michiel Pegtel

**Affiliations:** Laboratory for Orthopedic Pathophysiology and Regenerative Medicine, Istituto Ortopedico Rizzoli, Bologna, 40136 Italy; Department of Pathology, Cancer Center Amsterdam, VU University Medical Center, De Boelelaan 1117, 1081 HV Amsterdam, The Netherlands; Department of Biological Stress Response, Netherlands Cancer Institute, 1066 CX Amsterdam, The Netherlands; CNR—National Research Council of Italy, IGM, Bologna, 40136 Italy; SC Laboratory of Musculoskeletal Cell Biology, Istituto Ortopedico Rizzoli, Bologna, 40136 Italy

## Abstract

**Introduction:**

Administration of mesenchymal stem cells (MSCs) represents a promising treatment option for patients suffering from immunological and degenerative disorders. Accumulating evidence indicates that the healing effects of MSCs are mainly related to unique paracrine properties, opening opportunities for secretome-based therapies. Apart from soluble factors, MSCs release functional small RNAs via extracellular vesicles (EVs) that seem to convey essential features of MSCs. Here we set out to characterize the full small RNAome of MSC-produced exosomes.

**Methods:**

We set up a protocol for isolating exosomes released by early passage adipose- (ASC) and bone marrow-MSCs (BMSC) and characterized them via electron microscopy, protein analysis and small RNA-sequencing. We developed a bioinformatics pipeline to define the exosome-enclosed RNA species and performed the first complete small RNA characterization of BMSCs and ASCs and their corresponding exosomes in biological replicates.

**Results:**

Our analysis revealed that primary ASCs and BMSCs have highly similar small RNA expression profiles dominated by miRNAs and snoRNAs (together 64-71 %), of which 150–200 miRNAs are present at physiological levels. In contrast, the miRNA pool in MSC exosomes is only 2-5 % of the total small RNAome and is dominated by a minor subset of miRNAs. Nevertheless, the miRNAs in exosomes do not merely reflect the cellular content and a defined set of miRNAs are overrepresented in exosomes compared to the cell of origin. Moreover, multiple highly expressed miRNAs are precluded from exosomal sorting, consistent with the notion that these miRNAs are involved in functional repression of RNA targets. While ASC and BMSC exosomes are similar in RNA class distribution and composition, we observed striking differences in the sorting of evolutionary conserved tRNA species that seems associated with the differentiation status of MSCs, as defined by Sox2, POU5F1A/B and Nanog expression.

**Conclusions:**

We demonstrate that primary MSCs release small RNAs via exosomes, which are increasingly implicated in intercellular communications. tRNAs species, and in particular tRNA halves, are preferentially released and their specific sorting into exosomes is related to MSC tissue origin and stemness. These findings may help to understand how MSCs impact neighboring or distant cells with possible consequences for their therapeutic usage.

**Electronic supplementary material:**

The online version of this article (doi:10.1186/s13287-015-0116-z) contains supplementary material, which is available to authorized users.

## Introduction

Mesenchymal stem cells (MSCs) are intensively studied because they exhibit unique biological properties in vivo that are exploited for the treatment of many pathological conditions, most notably bone defects, degenerative illnesses, and autoimmunity [1].

MSCs are adult multipotent stem cells with self-renewal potential [2] that can differentiate into alternate phenotypes of the mesenchymal germ layer, namely osteoblasts, chondrocytes, and adipocytes [3]. The most common source of MSCs is the bone marrow [4, 5]; however, MSCs reside in many other tissues, notably adipose tissue, which is highly relevant because it is an easy accessible abundant source of stem cells [6]. Whether MSCs from different sources can be considered as the same cell type and whether distinct environments may influence their phenotype and function are still under debate [7, 8].

Recent advances suggest that the beneficial effects of MSCs derive from secreted factors rather than from their tissue intercalation and differentiation. The MSC secretome drives organ healing by inducing a shift from proinflammatory to anti-inflammatory cytokine production at the site of injury [9–11]. These observations support the development of cell-free, secretome-based therapies that circumvent the risks associated with stem cell-based therapies such as immune-mediated rejection, accumulation of genomic alterations, and senescence-induced genetic instability [12–14], and might require simpler safety regulations compared with their cell counterparts for clinical use [11].

The interest behind the MSC secretome goes beyond its application in tissue repair. Indeed, MSCs are strong contributors to tumor growth and progression in different cancer types [15–17], although anti-tumor activities have also been reported underscoring their pleiotropic properties [18, 19]. A unique aspect of MSCs is that they strongly respond to inflammatory signals causing homing to active tumor sites, where they provide paracrine survival, proangiogenic and immune-modulatory signals, similar to those that promote wound healing. Previous studies have focused on characterizing MSC-produced soluble factors (i.e. cytokines, chemokines, and growth factors). However, it is now clear that, in addition to soluble factors, extracellular vesicles (EVs) are a key instrument in cell–cell communication [20]. Among the many subtypes of EVs, endosome-derived exosomes have emerged as physiologically relevant and powerful components of the MSC secretome [11, 12, 21].

Exosomes are nano-sized EVs with remarkable physiological properties, originating through inward budding of the limiting membrane of late endosomes called multivesicular bodies (MVBs). Upon fusion of MVBs with the plasma membrane, exosomes are released into the extracellular milieu and can be either taken up by target cells residing in the microenvironment or carried to distant sites via biological fluids. Besides transporting characteristic protein and lipid signatures, exosomes package nucleic acids, most notably various RNA species with regulatory functions [22]. Arguably the most studied class of exosome-enclosed RNAs is the class of microRNAs (miRNAs), which function in repressing their target mRNAs in recipient cells in vitro [23–26] and in vivo [27]. However, we recently showed in B cells that miRNAs only account for a fraction of the exosomal RNA. Indeed, other noncoding transcripts, including repeats and structural RNAs, complete the exosomal RNA repertoire produced in B cells but also in other cell types [28, 29].

MSC-derived vesicles own remarkable properties typical of functional MSCs. Kordelas et al. [11] demonstrated the clinical efficacy of MSC exosomes to treat therapy-refractory graft-versus-host disease. Moreover, MSC-EVs regulate neurite outgrowth [30], promote angiogenesis both in vitro and in vivo [31], reduce myocardial ischemia/reperfusion injury [21], and repair acute kidney injury [32, 33]. Thus, it is reasonable to postulate that MSC-EVs transport key MSC-associated molecules which change the physiology of target cells in a specific manner. Proteomic analysis suggests that MSC-EVs or subclasses thereof contain critical surface markers and signaling molecules characteristic of the MSCs [34]. Moreover, prior quantitative PCR profiling analysis [35] showed that while some miRNAs are present both in MSCs and in their corresponding microvesicles, others are selectively represented.

To optimally understand and exploit the clinical potential of adult MSC-derived exosomes, it is important to define the relevant functional molecules they enclose. Comprehensive information on the complete RNA content of MSC exosomes is currently not available, and whether adult MSCs from different sources share similar small RNA repertoires or whether their content is different remains unknown.

Here we describe the first comprehensive deep-sequencing analysis of the small RNA profile of exosomes released by adult MSCs from two different sources: adipose-derived MSCs (ASCs) and bone marrow-derived MSCs (BMSCs). Our analysis of the exosomal content is useful for understanding how MSCs impact their microenvironment in resident niches and upon homing to damaged and inflamed tissues, which may have consequences for their therapeutic usage.

## Materials and methods

### Cell culture

Human adipose tissue samples from elective plastic surgery were obtained from the Department of Plastic Surgery of Tergooi Hospital after the approval of the Medical Ethical Committee of the VUmc (METC, Amsterdam, the Netherlands) and written informed consent. Adipose tissue was processed within 24 hours as described previously [36]. Briefly, adipose tissue was minced using a surgical scalpel and digested with 0.1 % collagenase A (Roche Mannheim, Germany) in phosphate-buffered saline (PBS) 1 % bovine serum albumin (BSA; Roche Diagnostics) under continuous shaking for 45 minutes at 37 °C. After Ficoll density separation (Lymphoprep; Axis-Shield, Oslo, Norway) cells were seeded at a density of 100,000 cells/cm^2^. The bone marrow of patients undergoing hip replacement was obtained from the Rizzoli Orthopedic Institute after the approval of the Comitato Etico dell’Istituto Ortopedico Rizzoli (Bologna, Italy) and written informed consent. Mononuclear cells were isolated by Ficoll Hystopaque gradient (Sigma-Aldrich, Milan, Italy) and seeded at a density of 250,000 cells/cm^2^. After 4 days, nonadherent cells were removed and fresh medium was added to the cultures. ASCs and BMSCs were expanded in alpha-minimum essential medium (α-MEM; Lonza, Breda, The Netherlands) containing 100 U/ml penicillin, 100 μg/ml streptomycin (Gibco, Bleiswijk, the Netherlands), and 10 % fetal bovine serum (FBS) or 5 % platelet lysate (PL) [37] and 10 U/ml heparin (Leo Pharma, Amsterdam, the Netherlands), in a humidified atmosphere of 5 % CO_2_ at 37 °C. The expression of typical MSC surface markers was analyzed by fluorescence-activated cell sorting (FACS), and the ability of the MSCs to undergo osteogenic differentiation was assessed by Alizarin red staining upon induction with ascorbic acid, dexamethasone, and β-glycerophosphate [38].

### Exosome isolation

MSC exosomes were collected from approximately 3.2 × 10^7^ cells at early passages (passages 2–3). Once MSC cultures reached 70 % confluence, cells were cultured for 24–48 hours in α-MEM containing exosome-depleted FBS or PL. Exosome-depleted FBS and PL were obtained by overnight centrifugation at 70,000 × *g* at 4 °C. Exosomes were isolated as described previously [39]. Briefly, MSC conditioned medium was centrifuged twice at 500× *g* for 10 minutes, twice at 2000 × *g* for 15 minutes and twice at 10,000 × *g* for 30 minutes. The supernatant was then transferred to Ultra-Clear tubes and centrifuged at 70,000 × *g* for 1 hour at 4 °C in a SW32Ti rotor (Beckman Coulter Inc., Woerden, The Netherlands). The exosome-containing pellet was washed with PBS and centrifuged at 70,000 × *g* for 1 hour. The pellet was then carefully resuspended in 200 μl PBS and used immediately or stored at −80 °C.

### Confocal laser scanning microscopy

For confocal laser scanning microscopy analysis, MSCs were seeded on poly-l-lysine-coated (Sigma-Aldrich) coverslips, fixed with 4 % paraformaldehyde, permeabilized with 0.1 % Triton-X 100 and blocked with PBS 10 % FBS (30 minutes). Slides were incubated with the primary antibodies against CD63 (BD Biosciences, Breda, The Netherlands) or EEA1 (Cell Signaling, Leiden, The Netherlands) and then with rabbit anti-mouse fluorescein isothiocyanate (FITC) antibody (DAKO, Heverlee, Belgium) for 30 minutes at room temperature. LysoTracker red (Molecular Probes, Bleiswijk, The Netherlands) was incubated with living cells before fixation. All stainings were imaged with a Leica DMRB microscope (Leica, Son, The Netherlands). Images were obtained through sequential scanning with the pinhole set at 1AE (standard). Fluorophores were excited using 488 nm (FITC) and 561 nm (Alexa594) laser lines.

### Western blotting

For western blot analysis, cells were lysed with RIPA buffer containing protease inhibitor cocktail (Roche), and the protein concentration was determined by BCA assay (Pierce, Etten-Leur, The Netherlands). Cell lysates and exosome preparations diluted in sample buffer were run on a 10 % SDS gel and blotted on a nitrocellulose membrane (GE Healthcare, Eindhoven, The Netherlands). Membranes were incubated with monoclonal antibodies against CD63, CD81, or cytochrome C (BD Biosciences) and horseradish peroxidase-conjugated rabbit anti-mouse secondary antibody (DAKO). Gels for CD63 and CD81 detection were run under nonreducing conditions.

### Transmission electron microscopy

Cell pellets were fixed with 2.5 % glutaraldehyde (Merck KGaA, Darmstadt, Germany) in phosphate buffer for 2 hours, post fixed with 1 % osmium tetroxide, dehydrated in a graded series of ethanol, and embedded in Epon (Electron Microscopy Sciences, Hatfield, PA,USA). Ultrathin sections were stained with uranyl acetate and lead citrate. Exosome preparations were mixed with an equal volume of 4 % paraformaldehyde (Sigma) in phosphate buffer. Then 5 μl solution were deposited on 200 mesh Formvar-carbon-coated electron microscopy (EM) nickel grids and left to adsorb for 20 minutes at room temperature. Samples were fixed with 1 % glutaraldehyde (Merck) in phosphate buffer, contrasted with uranyl oxalate (pH 7.0), and embedded in a mixture of 4 % uranyl acetate and 2 % methyl cellulose (25 cps; Sigma) in a 1:9 ratio on ice. Grids were then removed with stainless steel loops and the excess fluid was blotted with filter paper to ensure an appropriate thickness of the methyl cellulose film. After drying, grids were examined with a Zeiss EM109 transmission electron microscope (Zeiss, Oberkochen, Germany). Images were captured using a Nikon digital camera Dmx 1200F (Nikon Corporation, Tokyo, Japan), and ACT-1 software (Nikon Corporation).

### RNA isolation, RT-PCR, and generation of libraries for small RNA sequencing

Total RNA was isolated using Trizol Reagent (Invitrogen, Breda, The Netherlands) as described previously [39]. Exosome preparations were pretreated with RNase A (Sigma-Aldrich) at a final concentration of 400 ng/μl at 37 °C for 1 hour to degrade unprotected RNAs. The RNA quantity and purity were assessed with the Agilent 2100 Bioanalyzer system (Agilent, Amstelveen, The Netherlands). The expression analyses of differentiation and stemness-related genes were carried out using SYBR Green PCR master mix (Roche) in a LightCycler 480 real-time PCR system (Roche). Results were normalized with respect to glyceraldehyde 3-phosphate dehydrogenase (GAPDH) according to the ΔΔCt method [40]. cDNA libraries for sequencing were prepared using the TruSeq Small RNA Sample Preparation Kit (Illumina, Eindhoven, The Netherlands) following the manufacturer’s instructions. Amplified cDNA constructs were purified on 6 % PAGE gel and DNA molecules corresponding to 15–90 nucleotide transcripts were excised, eluted from gel, and concentrated by ethanol precipitation. Libraries were validated on the Bioanalyzer using the High Sensitivity DNA Chip (Agilent) and equimolarly pooled for the sequencing run. Sequencing was performed on a HiSeq 2000 (Illumina) paired end 100 cycle (PE100) run.

### Assignment of features to reads

Adapter sequences were trimmed from the 3′ ends of raw data using cutadapt (v1.1) [41] and the parameters “-O 12 -e 0..25”. Trimmed reads were aligned to the human genome (build hg19) using bowtie (v2.0.6) [42], and multiple valid alignments per read were reported (up to 50) using the parameters “--seed 42 --gbar 100 -D 10 -R 2 -L 20 -N 0 -i C,1 -k 50 --score-min L,0,-0.4”. For each read, only the alignments with the best score were used in subsequent analyses.

Several sources of genome annotation were used. GENCODE v.15 [43] was used, but transcripts with a total length larger than 120 were excluded, as well as genes of type “sense_intronic”, “sense_overlapping”, and “miRNA”. For miRNAs, annotations from miRBase (v.19) [44] were used, for both primary and mature transcript annotations. tRNA annotations were obtained from GENCODE/tRNA and were supplemented with metadata from GtRNAdb [45]. piRNA annotations from the piRNA database [46] were used while collapsing overlapping annotations into clusters. Repeat annotations and metadata from RepeatMasker were obtained from the UCSC (University of California Santa Cruz) genome browser (27 March 2013) [47].

Using the (possibly multiple) alignments per read, and the annotations described before, the set of (possibly partially) overlapping genes was determined. In the case that the set of genes all belonged to a certain “featuretype” (i.e. RNA type), the featuretype was assigned to that read. In the case that the featuretype was ambiguous, the following steps were taken. If the length of the read was larger than 25 nucleotides, the featuretype “miRNA, processed” was eliminated; otherwise, the featuretype “miRNA, premature” was eliminated. If the length of the read was larger than 32 nucleotides, the featuretype “piRNA” was eliminated. If at this point the featuretype could be determined unambiguously, this featuretype was assigned. Otherwise, if there were two possible featuretypes, of which one was a repeat, the nonrepeat featuretype was assigned to the read. If this was not the case, and the read had two or more possible alignments, all featuretypes which were indicated by only one alignment were eliminated. Again, if at this point the featuretype could be determined unambiguously, this featuretype was assigned. In other cases, where the featuretype was not determined unambiguously, the read was designated as “Ambiguous” (Figure S1 in Additional file 1).

For further analyses, the alignments were processed and tables enumerating the featuretype, unique sequence, and count were created. This process enabled the analyses of sequence lengths split out by featuretype, and the analyses of fractions of RNA types per sample. For the analysis of differential expression of specific miRNA, the featuretype of miRNAs was further refined to resolve the miRNA name. Analogously, for detailed analysis of tRNAs and repeats, the featuretypes of these two classes were further refined to include the tRNA anti-codon and repeat family, respectively.

### Correlations of samples

Correlations were only performed on genes which had at least a total number of 15 reads over the cell samples, or five reads over the exosome samples. Counts were normalized using the trimmed-mean-of-M-values normalization. Log values were calculated, and log values over zero were imputed per sample, by taking the lowest nonzero normalized value, dividing by two, and taking the log of that value (i.e. zero counts were imputed by an estimation of the lower limit of detection). Pearson correlation coefficients between the samples were calculated. In heatmaps, genes were clustered by their standardized euclidean distance, and samples were clustered by their correlation coefficients.

### tRNA/mRNA 3′ UTR complementarity analysis

A nonredundant list of tRNA fragment sequences was made from the 20 most abundant tRNA fragments per sample. A BLAST database of mRNA 3′ UTRs was prepared using GENCODE v15 transcript annotations (only for transcripts having a complete CDS annotation). tRNA fragment sequences were searched for complementarity to 3′ UTRs using BLAST (blastn v.2.2.25), with parameters allowing only ungapped, complementary hits (“-u T -S 2”), seed length 6, and an adjusted effective search space size which was set to the total genomic length spanned by the 3′ UTRs used in the database (to adjust for overlapping 3′ UTRs originating from the same genomic sequence). The maximum allowed *e* value was 1.0. For each hit, the conservation of the genomic region that matched to the tRNA was inspected using phyloP 100-way data (obtained from UCSC tracks, dd. 20MAR2015). Additionally, the conservation of the matching region relative to the shortest overlapping 3′ UTR was calculated.

### Statistical analyses

All statistical analyses were carried out in R software (http://www.r-project.org/). Differential expression was determined using the exactTest routine from the edgeR package [48] and common library dispersions. Counts were normalized using the supplied trimmed-mean-of-M-values algorithm, except in the analysis of high-level feature types (e.g. “miRNA”, “piRNA”, “repeat”), in which case total library sizes were used to normalize.

Research was carried out in compliance with the Helsinki Declaration and all experimental protocols were approved by the Ethical Committee of the VU University Medical Center and of the Rizzoli Orthopaedic Institute.

## Results

### Mesenchymal stem cells from adipose tissue and bone marrow release exosome-like EVs enriched in small RNAs

To study the mesenchymal stem cell-released EVs we isolated primary adult MSCs from human bone marrow (BMSCs, *n* = 4) and adipose tissue (ASCs, *n* = 3) (Figure S2 in Additional file 1). Early passage MSCs (passages 0–2) were expanded in exosome-depleted FBS or PL to support *in vitro* expansion [36].

**Fig. 1:**
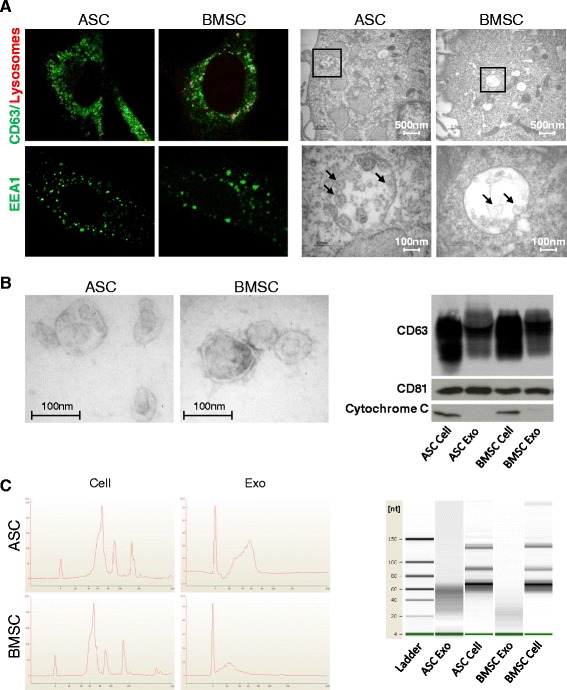
ASCs and BMSCs contain relatively few MVBs and release exosome-like vesicles enriched in small RNAs. **a** Immunofluorescent staining of CD63 (*top left*) and EEA1 (*bottom left*), and ultrastructure of MVB-like endosomes (*right*) in ASCs and BMSCs. **b** Transmission electron microscopy micrographs of exosomes isolated from ASCs and BMSCs (*left*); and western blot for CD63, CD81, and cytochrome C in cells and corresponding exosomes (*right*). **c** Bioanalyzer small RNA profile of cells and exosomes showing enrichment of 20–70 nucleotide small RNAs in exosomes. *ASC* adipose-derived mesenchymal stem cell, *BMSC* bone marrow-derived mesenchymal stem cell, *EEA1* early endosome antigen A1, *exo* exosome

The MSC endosomal compartment was analyzed by immunofluorescence and by EM. The immunofluorescent staining showed high punctate expression of CD63, which was mainly localized in nonacidic vesicles in the perinuclear region of the cells, as determined by lysotracker (Fig. 1a, top left). The early endosome antigen A1 (EEA1) staining highlighted the presence of numerous early endosomes distributed throughout the cell body (Fig. 1a, bottom left). However, despite the expression of endosomal markers, the ultrastructure of the stem cells revealed relatively low numbers of late endosomes with internal vesicular structures, suggestive of MVB-like compartments and/or secretory lysosomes. These compartments had a diameter of about 500 nm and enclosed 40–100 nm intraluminal vesicles (Fig. 1a, right). The low abundance of MVB-like organelles detectable by EM suggests that the high amount of CD63 observed in the perinuclear space of MSCs is associated with intracellular membranes not related to MVBs.

EVs were isolated by differential centrifugation as described previously [39] and their purity was confirmed by EM and western blotting for CD63 and CD81. Cytochrome C was assessed to exclude contamination by apoptotic bodies (Fig. 1b). Although differential centrifugation is the most commonly used method to isolate exosomes from culture supernatant [49], it is not possible to rule out the presence of other types of EVs in the exosome preparations when using this procedure. However, because our preparations obtained by ultracentrifugation seem to be enriched for exosomes in terms of size, shape, and tetraspanin content, we consider these MSC-EVs as “exosomes”. To exclude potential contamination of MSC-exosome preparations by PL or FBS-derived EVs, we analyzed CD63 and CD81 in exosome-depleted culture media subjected to differential centrifugation. Since no CD63 was detectable, we concluded that our preparations contained mainly MSC-derived exosomes (Figure S3A in Additional file 1).

To define the small RNA composition of the MSC exosomes we degraded any unprotected RNA in exosome preparations by adding exogenous RNase A. Subsequently, we isolated cellular and exosomal RNA, which was subjected to Bioanalyzer profiling. The small RNA profile of exosomes revealed characteristic peaks between 20 and 70 nucleotides, suggestive of the presence of miRNAs and tRNAs. The size distribution in cells was more heterogeneous and included longer transcripts (Fig. 1c). We constructed cDNA libraries of MSC cellular and exosomal RNA molecules with a length ranging between approximately 15 and 90 nucleotides (Figure S3B in Additional file 1).

We observed that early passage primary MSCs contain relatively few MVBs, which suggests that these cells secrete relatively few exosomes compared with other cell types [23, 50, 51]. However, MSC-released exosomes incorporate a small RNA population that is protected from exogenous RNases.

### Mesenchymal stem cells and their exosomes have a different RNA composition

**Fig. 2:**
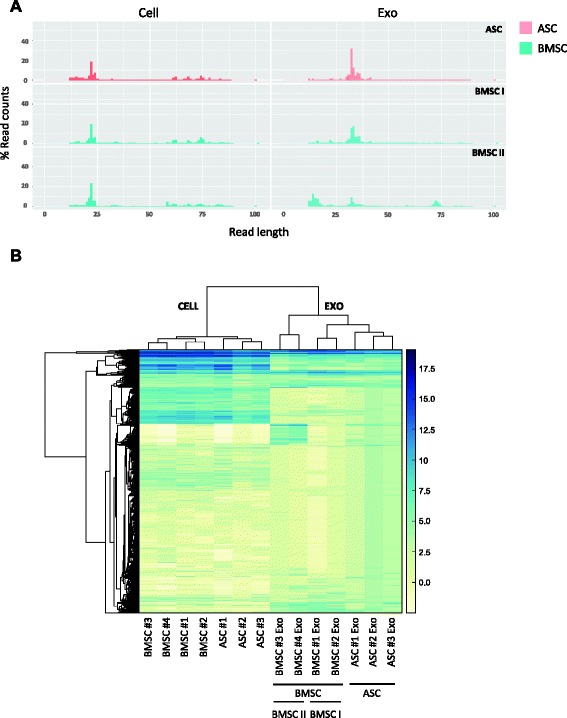
MSCs and their exosomes display a different small RNA composition. **a** Length distribution of RNAseq aligned reads in ASCs and BMSCs and corresponding exosomes (one representative donor). **b** Unsupervised hierarchical clustering analysis of MSCs and exosomes based on the total small RNA content. *ASC* adipose-derived mesenchymal stem cell, *BMSC* bone marrow-derived mesenchymal stem cell, *exo* exosome

**Fig. 3:**
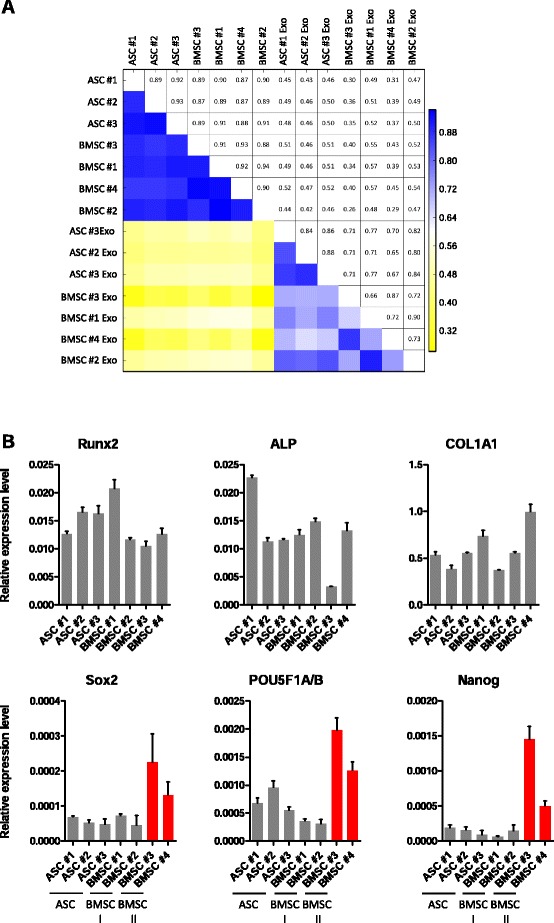
Exosomes released by MSCs at different stages of differentiation correlate moderately with each other. **a** Correlation matrix of MSC and exosome samples. **b** Relative expression levels of early osteogenic differentiation (*top*) and multipotency (*bottom*) genes analyzed by quantitative PCR. Data were normalized to GAPDH. BMSC II express higher levels of Sox2 (ANOVA test: *p* = 0.006; Fisher’s PLSD test: *p* = 0.02 BMSC II vs. ASC, *p* = 0.03 BMSC II vs. BMSC I), POU5F1A/B (ANOVA test: *p* = 0.01; Fisher’s PLSD test: *p* = 0.03 BMSC II vs. ASC, *p* = 0.01 BMSC II vs. BMSC I) and Nanog (ANOVA test: *p* = 0.03; Fisher’s PLSD test: *p* = 0.056 BMSC II vs. ASC) compared with the other subtypes. *ALP* alkaline phosphatase, *ANOVA* analysis of variance, *ASC* adipose-derived mesenchymal stem cell, *BMSC* bone marrow-derived mesenchymal stem cell, *COL1A1 *collagen type 1 alpha 1, *exo* exosome, *GAPDH* glyceraldehyde 3-phosphate dehydrogenase, Fisher's PLSD Fisher's Protected Least Significant Difference

The sequencing of the libraries yielded a total of 25 million reads. The cell samples showed a wide distribution of read length, with a predominant peak around 22 nucleotides (Fig. 2a), regardless of the tissue origin and donor. Exosomes released by ASCs had a major peak between 31 and 36 nucleotides, while BMSC exosome samples showed two different profiles and were therefore classified into subtypes (BMSC I and BMSC II). BMSC I exosomes had a length distribution similar to that of ASC, while BMSC II exosomes displayed additional peaks at 15 and 70 nucleotides. In order to assess the variability among samples we performed an unsupervised hierarchical clustering analysis. Clustering analysis revealed a strong similarity among MSC samples irrespective of the stem cell source (Fig. 2b). Interestingly, however, when looking at the exosome preparations, ASC samples cluster tightly together, while BMSC I and BMSC II exosome samples appear dissimilar from each other. In accordance, the correlation analysis (Fig. 3a) indicated a strong correlation among cellular samples (0.87 <*r* <0.94). This correlation decreased when comparing exosome libraries: the Pearson coefficient within the BMSC I and BMSC II subtypes was 0.90 and 0.87 respectively, while it ranged from 0.66 and 0.73 between the two subgroups. Among ASC exosomes the coefficient ranged from 0.84 to 0.88, while collectively was between 0.65 and 0.84 between ASC and BMSC exosomes.

The relatively low correlation between the two subtypes of BMSC exosomes prompted us to investigate whether the producing cells may represent different differentiation stages. Because MSCs can spontaneously undergo osteogenic differentiation during in-vitro expansion [52], we analyzed the expression of early osteogenic markers—i.e. Runx2, alkaline phosphatase (ALP), and collagen type 1 alpha 1 (COL1A1)—and of the stemness-related genes SOX2, POU5F1A/B, and Nanog by quantitative PCR (Fig. 3b). While we did not observe differences in the expression of bone-related genes, BMSC II subtype cells had elevated expression of Sox2, POU5F1A/B, and Nanog as compared with the other subtypes (ANOVA test, Fisher’s PLSD correction: *p* <0.05 BMSC II vs. ASC and BMSC II vs. BMSC I for Sox2, and POU5F1A/B, and BMSC II vs. ASC for Nanog). The different expression levels of the pluripotency genes reflecting the stemness of the MSCs might explain the dissimilarity in exosome small RNA composition.

**Fig. 4:**
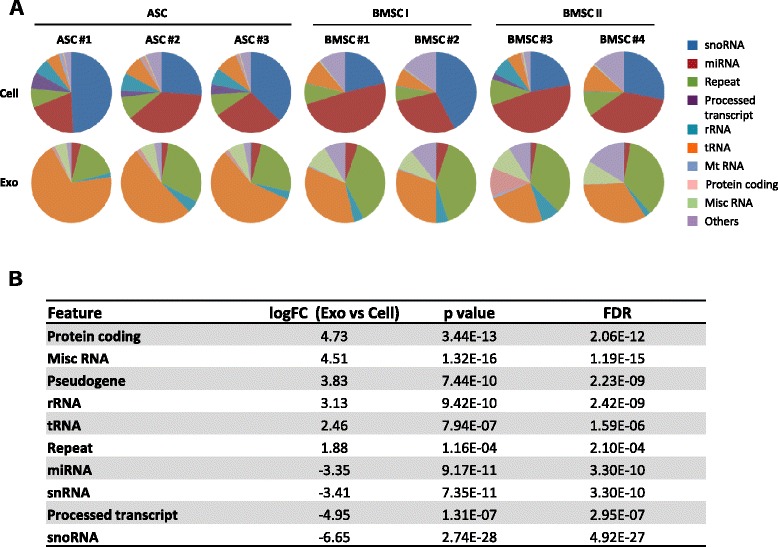
Mesenchymal stem cells and their exosomes have a different RNA class distribution. **a** Relative distribution of overrepresented RNA species in MSCs and exosomes. **b** Differential representation of RNA classes in MSC exosomes versus cells ordered by logFC. *ASC* adipose-derived mesenchymal stem cell, *BMSC* bone marrow-derived mesenchymal stem cell, *exo* exosome, snoRNA small nucleolar RNA, logFC log fold-change, FDR false discovery rate

The correlation matrix analysis (Fig. 3a) highlighted a weak correlation between cells and corresponding exosomes (*r* ≤0.57). This implies that exosomes do not strictly reflect the RNA composition of the cells of origin, but selectively incorporate a variety of RNA species. Indeed, we observed enrichment of distinct RNA classes in cells while others were overrepresented in exosomes (Fig. 4a, b). In all MSC samples, miRNAs and small nucleolar RNAs (snoRNAs) were the most abundant classes of RNA in the cells, although their proportion was variable independently of the tissue source (19–49 % and 21–49 %, respectively) (Fig. 4a). These two classes together accounted for 64–71 % of the entire cellular small RNA pool, followed by repeats (6–11 %), tRNA (5–11 %), and rRNAs (up to 8 %). In contrast, exosome libraries were highly enriched in the class of tRNAs, which represented >50 % of total small RNAs in adipose-derived exosomes and 23–35 % in bone marrow exosomes, and repeats, ranging from 17–30 % in adipose exosomes to 24–40 % in bone marrow exosomes (Fig. 4a). The dominance of tRNAs in exosomes is consistent with their function and abundance in the cellular cytoplasm. Other represented classes in exosomes were miscellaneous RNAs, rRNAs, and miRNAs, the latter representing only 2–5 % of the small RNA repertoire. In sharp contrast with that observed in MSC libraries, snoRNAs only represented a very small proportion of the exosomal RNA content (<0.6 %).

In summary, our data indicate that miRNAs and snoRNAs are significantly enriched in the cells (miRNA: logFC 3.35, FDR 3.3 × 10^–10^; snoRNA: logFC 6.65, FDR 4.9 × 10^–27^) while tRNAs and repeats form a defined pool of RNAs heavily enriched in exosomes (tRNA: logFC 2.46, FDR 1.59 × 10^–6^; repeats: logFC 1.88, FDR 2.1 × 10^–4^), suggestive of preferential sorting and release. The length distribution (Fig. 2a) showed predominant peaks between 31 and 36 nucleotides in ASC and BMSC I exosomes and additional peaks at around 15 and 70 nucleotides in BMSC II exosomes, suggestive of the presence of full-length tRNA and tRNA fragments in exosomes. Since tRNAs and tRNA fragments are involved in translation regulation and RNA silencing, these observations may point to a physiological link between post-transcriptional regulation and exosome biogenesis in MSCs [53, 54].

### Mesenchymal stem cell exosomes selectively incorporate specific miRNAs

A substantial proportion of the cellular small RNA content in MSCs (19–49 %) is miRNA. However, this class is underrepresented in exosomes (2–5 % of the total small RNA) (Fig. 4a). Because of this discrepancy we investigated whether cells and exosomes share similar miRNA content.

**Fig. 5:**
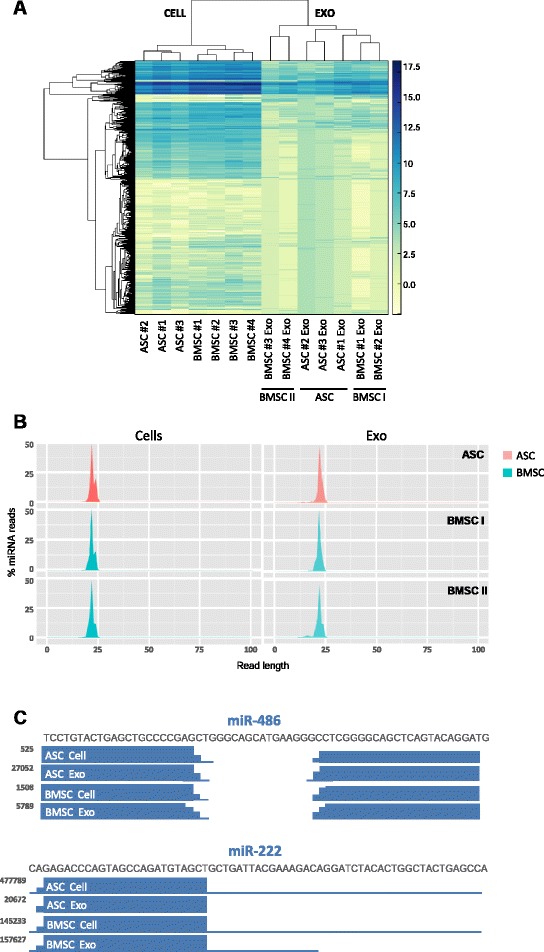
MSCs and their exosomes display a different miRNA repertoire. **a** Unsupervised hierarchical clustering analysis of MSCs and their exosomes based on the miRNA content. **b** Length distribution of miRNA reads in ASC and BMSC I and BMSC II cells and exosomes (one representative donor). **c** Sequence coverage of highly represented miRNA genes (based on UCSC genome browser custom tracks) showing a predominant presence of mature forms (one or both miRNA arms) in MSCs and exosomes. *Y* axis indicates the normalized counts (rpm). *ASC* adipose-derived mesenchymal stem cell, *BMSC* bone marrow-derived mesenchymal stem cell, *exo* exosome

In line with our previous findings on the total small RNA profile, unsupervised clustering analysis and correlation analysis based on the miRNA content only revealed high similarity within cellular samples (0.89 <*r* <0.96), while exosome samples displayed greater variability (0.75 <*r* <0.90). Overall, the correlation between cells and exosomes appears weak (Fig. 5a; and Figure S4A in Additional file 1).

To assess whether miRNAs were present as precursors or fully processed transcripts we looked at the length distribution of the miRNA reads (Fig. 5b), and found that in all MSC subtypes, both in exosomes and corresponding cells, the vast majority of miRNA sequences ranged between 20 and 25 nucleotides. Accordingly, the sequencing coverage of the most represented miRNA genes (UCSC genome browser) (Fig. 5c) predominantly shows the presence of mature miRNAs mapping to the 5p and/or 3p arms of the precursor.

**Fig. 6:**
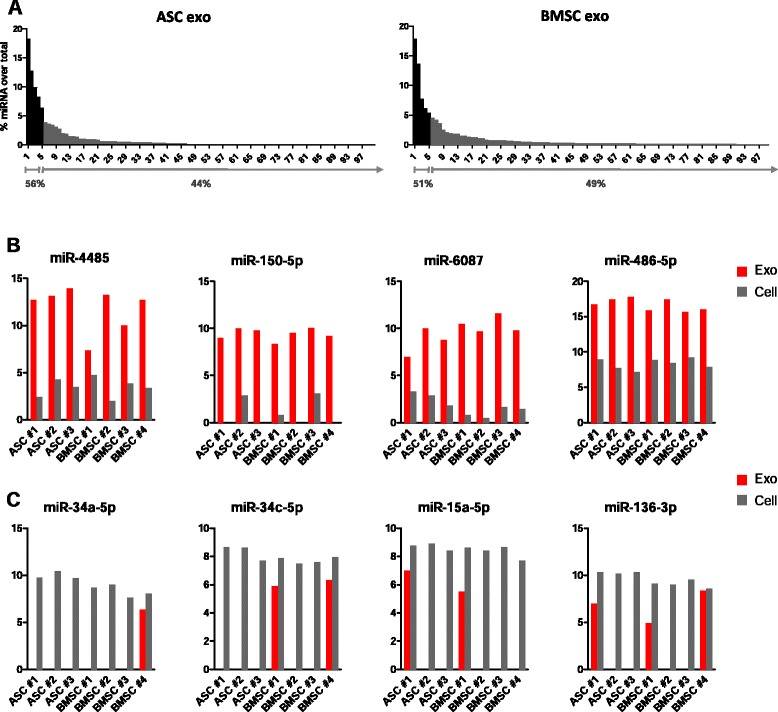
MSC exosomes selectively incorporate specific miRNAs. **a** Relative proportion of miRNAs in the repertoire of total miRNA reads. The five most abundant miRNAs represent 50 % of the total miRNA reads. **b** Overrepresented and **c** underrepresented miRNAs in MSC exosomes as compared with producing cells (LogFC >3; FDR <0.0002). *ASC* adipose-derived mesenchymal stem cell, *BMSC* bone marrow-derived mesenchymal stem cell, *exo* exosome

We then examined the relative proportion of individual miRNAs in the repertoire of total miRNA reads (Fig. 6a; and Figure S4B in Additional file 1). Surprisingly, the five most abundant miRNAs (miR-486-5p, miR-10a-5p, miR-10b-5p, miR-191-5p, and miR-222-3p in ASC exosomes; and miR-143-3p, miR-10b-5p, miR-486-5p, miR-22-3p, and miR-21-5p in BMSC exosomes) accounted for 43–59 % of the total miRNA reads. To evaluate the relative distribution of miRNAs in cells and exosomes, we ranked cellular and exosomal miRNA based on the reads per million (rpm) values and compared the 20 most represented miRNAs in cells and exosomes (Table 1). miR-21-5p, miR-22-3p, miR-10b-5p, and miR-222-3p were among the most represented in both cells and exosomes; however, various miRNAs (shown in bold in Table 1) were only present either in the list of cellular or in the list of exosomal highly represented miRNAs. We next asked whether specific miRNAs may be preferentially excreted or retained in the cells. Figure 6b shows the top four miRNAs overrepresented in exosomes compared with MSCs (logFC >7; FDR <5 × 10^–15^). On the other hand, miR-34a-5p, miR-34c-5p, miR-15a-5p, and miR-136-3p were significantly overrepresented in cells compared with exosomes (logFC >3; FDR <3 × 10^–6^) (Fig. 6c). The relative abundance of these miRNAs is shown in Figure S4C in Additional file 1. Altogether, the nonrandom distribution of miRNAs is consistent with a sorting mechanism for disposal of “unused” small RNAs or for communication with the surrounding environment as shown in other cell types [55].

**Table 1:** Most abundant miRNAs in MSC exosomes and cells

Rank	Most represented miRNA in exosomes		Most represented miRNA in cells	
	miRNA	rpm ASC exosomes	miRNA	rpm ASC cells
1	**miR-486-5p**	**172,837**	miR-21-5p	145,332
2	miR-10a-5p	117,453	miR-22-3p	92,902
3	miR-10b-5p	104,447	miR-10b-5p	82,089
4	**miR-191-5p**	**92,545**	miR-222-3p	74,725
5	miR-222-3p	49,405	miR-143-3p	73,794
6	miR-22-3p	37,205	let-7a-5p	71,686
7	let-7a-5p	28,814	miR-10a-5p	44,303
8	miR-21-5p	27,233	miR-92a-3p	33,827
9	miR-127-3p	27,071	let-7f-5p	33,248
10	miR-143-3p	22,579	**let-7i-5p**	**22,624**
11	**miR-99b-5p**	**21,471**	**miR-127-3p**	**17,303**
12	miR-100-5p	18,836	**miR-148a-3p**	**17,109**
13	miR-92a-3p	18,217	miR-26a-5p	16,127
14	let-7f-5p	17,589	miR-92b-3p	14,852
15	miR-92b-3p	16,124	**miR-21-3p**	**14,548**
16	miR-26a-5p	13,207	**miR-221-3p**	**12,481**
17	**miR-146a-5p**	**12,212**	**miR-16-5p**	**11,544**
18	**miR-4485**	**10,656**	miR-100-5p	11,180
19	**miR-146b-5p**	**10,,558**	**miR-31-5p**	**9521**
20	**miR-151a-3p**	**10,262**	**miR-411-5p**	**8051**
	miRNA	rpm BMSC exosomes	miRNA	rpm BMSC cells
1	miR-143-3p	124,950	miR-143-3p	185,884
2	miR-10b-5p	103,485	miR-21-5p	150,993
3	**miR-486-5p**	**91,274**	miR-22-3p	105,358
4	miR-22-3p	74,730	let-7a-5p	91,387
5	miR-21-5p	47,445	miR-10b-5p	42,729
6	miR-222-3p	46,094	miR-222-3p	38,076
7	miR-191-5p	45,054	miR-27b-3p	35,496
8	miR-100-5p	41,668	let-7f-5p	29,054
9	let-7a-5p	38,486	let-7i-5p	21,993
10	**miR-99b-5p**	**29,011**	**miR-26a-5p**	**21,934**
11	miR-92a-3p	24,941	miR-100-5p	21,157
12	miR-127-3p	21,319	miR-127-3p	14,836
13	let-7f-5p	21,203	**miR-148a-3p**	**12,789**
14	miR-92b-3p	20,938	miR-92b-3p	12,306
15	**miR-423-5p**	**19,807**	miR-92a-3p	11,840
16	**miR-10a-5p**	**14,716**	miR-191-5p	11,384
17	miR-27b-3p	13,604	**miR-21-3p**	**11,372**
18	let-7i-5p	11,997	**miR-125b-5p**	**10,699**
19	**miR-28-3p**	**10,554**	**let-7b-5p**	**9603**
20	**miR-125b-5p**	**10,378**	**miR-16-5p**	**9583**

miRNAs present only in the list of cellular or in the list of exosomal miRNAs are highlighted in bold

*ASC* adipose-derived mesenchymal stem cell, *BMSC* bone marrow-derived mesenchymal stem cell, *miRNA* microRNA, *MSC* mesenchymal stem cell, *rpm* reads per million

### tRNA-derived RNA fragments are highly represented in MSC exosomes

The dramatic overrepresentation of tRNA sequences in MSC exosomes prompted us to investigate which tRNAs were the most represented and whether these could be functional processed transcripts or degradation products.

**Fig. 7:**
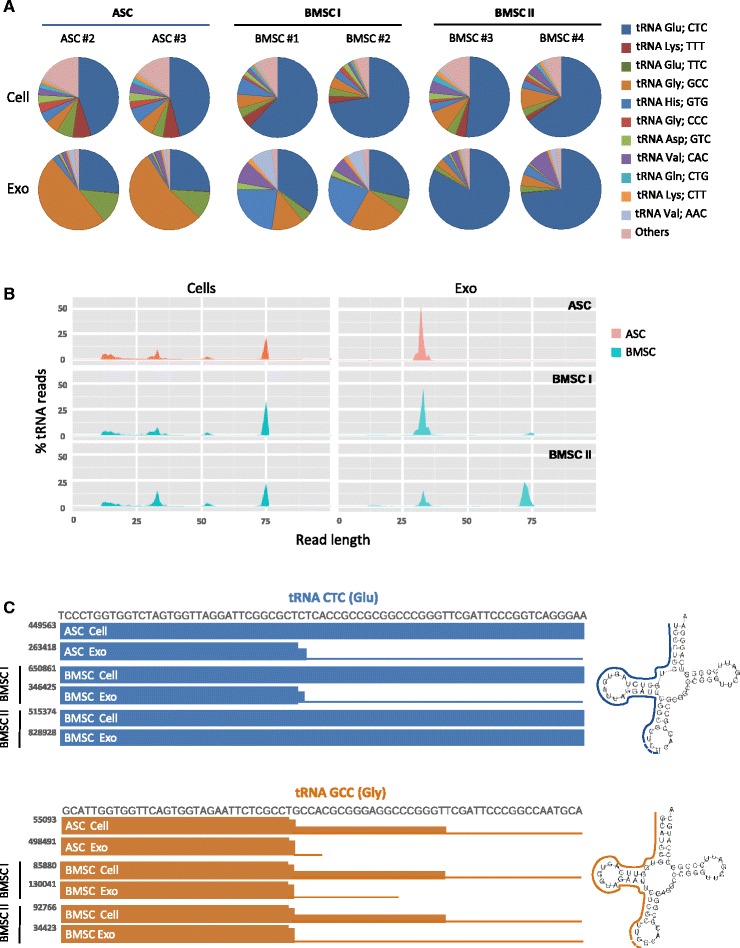
tRNA-derived RNA fragments are highly represented in MSC exosomes. **a** Relative distribution of highly represented tRNAs in MSCs and respective exosomes. **b** Length distribution of tRNA sequencing reads in ASC and BMSC I and II cells and exosomes. **c** Sequence coverage of highly represented tRNA genes (based on UCSC genome browser custom tracks). *Y* axis indicates the normalized counts (rpm). *ASC* adipose-derived mesenchymal stem cell, *BMSC* bone marrow-derived mesenchymal stem cell, *exo* exosome

Our analysis revealed that generally the adult MSC subtypes have similar tRNA profiles irrespective of the tissue source (Fig. 7a, top). tRNA CTC (Glu) was highly represented both in ASCs and in BMSCs, accounting for 43–72 % of the total tRNA reads. In order to exclude potential experimental biases leading to high representation of one specific tRNA sequence, we also analyzed the tRNA profile obtained by small RNAseq in an unrelated cell type—i.e. lymphoblastoid cells (LCLs) (Figure S5A in Additional file 1). Interestingly, we found a very distinct tRNA distribution in these cells suggesting that, similar to miRNAs, tRNA profiles may be useful as indicators of tissue origin. Overall, exosomal tRNA profiles appeared distinct from the cellular profiles (Figure S6 in Additional file 1) and displayed more intergroup variability (Fig. 7a, bottom). The five most prevalent tRNA sequences in MSC exosomes accounted for 87–97 % of the total exosomal tRNA pool (consisting of 40–54 different acceptors). Interestingly, while in BMSCs and LCLs the most abundant tRNAs in cells and exosomes clearly correspond, the most abundant tRNA in ASC exosomes, tRNA GCC (Gly), only represented a small fraction (5 %) of the total cellular tRNA (Fig. 7a). Moreover, this tRNA was overrepresented in ASC exosomes compared with BMSC exosomes (logFC 3.8; FDR 1.1 × 10^–7^) (Figure S5B in Additional file 1).

To investigate whether MSC libraries contain full-length tRNAs or processed transcripts we analyzed the length distribution of the tRNA reads (Fig. 7b). Surprisingly, both in cells and exosomes, tRNA fragments constitute a considerable fraction of the total tRNA. Although MSCs from both tissue origins display a broad range of tRNA lengths, exosomal tRNA sequences have specific fragment sizes. ASC exosomes and BMSC I exosomes mainly contained fragments of 30–35 nucleotides, whereas BMSC II exosomes also show a dominant peak at 70–75 nucleotides. Intrigued by these findings we zoomed into the genomic coverage and length distribution of the most represented tRNA genes (Fig. 7c; and Figure S5C in Additional file 1). Strikingly, while at the cellular level adipose and bone marrow MSCs contain the full-length form of the most abundant tRNA, tRNA CTC (Glu), exosomes released by ASC and BMSC I exosomes consistently display the 33-nucleotide 5′ halves of the most abundant sequences (Fig. 7c; and Figure S5C in Additional file 1). BMSC II exosomes that are produced by cells with high expression of pluripotency factors seem to preferentially enclose the full-length form of tRNA CTC (Glu), and the 33-nucleotide fragments of other abundant tRNA species (Figure S5C in Additional file 1). The remarkable differences in tRNA composition observed between adipose and bone marrow exosomes warrant future investigations into the paracrine effects of adult stem cells from different sources and at different stages of differentiation.

Although the function of tRNA fragments and tRNA halves in mammalian cells is still largely unknown, a miRNA/siRNA (small interfering RNA)-like function for these tRNA species has been recently suggested [56]. Here we performed a bioinformatic analysis to evaluate the antisense complementarity of the most abundant tRNA species in cells and exosomes to the 3′ UTRs of annotated protein-coding transcripts. We found a group of putative candidate targets (Table 2; and Additional file 2), many of which have undefined biological function. Interestingly, among the potential targets we found factors involved in stem cell self-renewal and MSC differentiation, such as TFCP2L1, RUNX2, and SOX11, the transforming growth factor-beta (TGFβ) signaling mediator SMAD3, and immune-related factors such as HHLA2, EMR2, and TRIM62.

**Table 2:** Complementarity analysis of tRNA species to 3′ untranslated regions of protein-coding genes

Query	Query sequence	Hit length	*e* value	Bitscore	Gene name	Conservation value	Normalized conservation value
tRNA; Glu; CTC	AGTGGTTAGGATTCGGCGCTCTCACCGCCGCGGCCC	14	0.19	28.2	GOLGA6A	0.510	0.970
tRNA; Glu; TTC	TCCCTGGTGGTCTAGTGGCTAGGATTCGGCGCTTT	24	0.012	32.2	TFCP2L1	0.146	1.067
		14	0.19	28.2	NELF	-0.303	0.962
		17	0.74	26.3	KIAA0513	-0.367	1.013
		13	0.74	26.3	ZBTB45	-0.447	0.950
		13	0.74	26.3	HHIPL1	-1.103	0.808
		13	0.74	26.3	HSP90AA1	-0.248	0.982
		13	0.74	26.3	TULP4	1.937	1.345
		13	0.74	26.3	SMAD3	0.259	1.001
		13	0.74	26.3	RPS19	-0.474	0.984
		13	0.74	26.3	ZNF662	0.140	0.990
		13	0.74	26.3	AC104841.2	2.810	1.542
tRNA; Gly; GCC	GCATGGGTGGTTCAGTGGGAGAATTCTCGCCT	18	0.19	28.2	PHF13	0.042	0.914
		14	0.19	28.2	KDSR	-0.243	0.904
		14	0.19	28.2	PLAGL2	0.634	0.881
		13	0.74	26.3	NOX5	-0.231	0.982
		13	0.74	26.3	EPM2AIP1	0.373	0.996
		13	0.74	26.3	GTF3C1	-0.476	1.095
		13	0.74	26.3	GPR110	0.293	1.053
		13	0.74	26.3	TPI1	0.241	0.971
		13	0.74	26.3	PPP2R1B	-0.302	0.875
		13	0.74	26.3	LUZP2	0.125	0.998
		13	0.74	26.3	VPS41	-0.243	0.977
		13	0.74	26.3	SSR1	0.187	1.025
		13	0.74	26.3	RUNX2	1.358	0.956
		13	0.74	26.3	MAP1LC3B	-0.254	0.890
	GCATGGGTGGTTCAGTGGTAGAATTCTCGCCG	14	0.19	28.2	GCM1	0.127	1.032
		17	0.74	26.3	SLC2A13	0.507	0.983
		13	0.74	26.3	GTF3C1	-0.476	1.095
		13	0.74	26.3	HHLA2	-0.075	1.004
		13	0.74	26.3	TPI1	0.241	0.971
		13	0.74	26.3	PEG10	-0.126	0.806
		13	0.74	26.3	EMR2	-0.166	0.954
		13	0.74	26.3	SSR1	0.187	1.025
		11	0.7	22.3	THAP5	0.155	0.979
		11	0.7	22.3	THAP5	0.393	1.029
	GCATGGGTGGTTCAGTGGTAGAATTCTCGCCTG	14	0.19	28.2	GCM1	0.127	1.032
		17	0.74	26.3	SLC2A13	0.507	0.983
		13	0.74	26.3	GTF3C1	-0.476	1.095
		13	0.74	26.3	HHLA2	-0.075	1.004
		13	0.74	26.3	TPI1	0.241	0.971
		13	0.74	26.3	PEG10	-0.126	0.806
		13	0.74	26.3	EMR2	-0.166	0.954
		13	0.74	26.3	SSR1	0.187	1.025
		11	0.73	22.3	THAP5	0.155	0.979
		11	0.73	22.3	THAP5	0.393	1.029
	GCATTGGTGGTTCAGTGGTAGAATTCTCGCCTGCCACGCGGGAGGCCCGGGT	20	0.012	32.2	SOX11	0.180	0.752
		14	0.19	28.2	GCM1	0.127	1.032
		17	0.74	26.3	SLC2A13	0.507	0.983
		17	0.74	26.3	FAM57B	-0.158	0.849
		13	0.74	26.3	HHLA2	-0.075	1.004
		13	0.74	26.3	C1orf159	-0.663	0.984
		13	0.74	26.3	KLC2	-0.224	0.835
		13	0.74	26.3	PEG10	-0.126	0.806
		13	0.74	26.3	DRG2	-0.468	0.817
		13	0.74	26.3	VPRBP	1.514	0.913
		13	0.74	26.3	TRIM62	-0.098	0.921
		13	0.74	26.3	EMR2	-0.166	0.954
		13	0.74	26.3	ANXA8L2	2.926	1.223

## Discussion

The administration of MSC-EVs is advantageous over cell-based therapy because it eliminates the safety concerns associated with the injection of multipotent cells into patients [12]. MSC-secreted exosomes have recently been shown to improve therapy-refractory graft-versus-host disease [11] and to promote organ healing in various preclinical models [21, 32, 33]. Comprehensive characterization of these vesicles is therefore a critical step in understanding their biological activity to maximize clinical utility.

MSCs have been described before as prolific producers of exosomes when compared with some other cell types [57]. However, this conclusion was based on the use of myc-transformed (immortalized) human embryonic stem cell-derived MSCs [58], which was required to overcome the limited in-vitro expansion potential. Because immortalized cells may secrete pro-oncogenic material via EVs [59], the clinical usefulness of these vesicles is uncertain. In addition, immortalized MSCs may produce EVs with an altered content, casting additional doubt as to whether these EVs are representative of their natural counterparts [60]. We show here that unmanipulated MSCs in culture produce few EVs with exosome characteristics. Accordingly, MSCs in culture possess relatively few prototypical MVBs compared with most cell types analyzed thus far in our laboratory [23, 50]. Although MSC exosomes have the same morphology as exosomes from B-cell blasts and carry typical marker proteins, they may differ in compartmentalization, biogenesis, and therefore RNA composition. Moreover, we presume that factors such as tissue origin (adult or embryonic) and stemness could influence both exosome production and content.

The very low intragroup and intergroup variability among MSC samples indicates that donor-specific characteristics and the tissue-specific microenvironment do not significantly influence the small RNA expression profile of the cells. However, important differences emerged when comparing EV preparations. The variability between ASC and BMSC EVs suggests that the tissue-specific microenvironment might influence the exosomal sorting of the MSCs. The intragroup variability indicates that cell-intrinsic factors, such as the differentiation status of the cells, might dictate which signals are conferred by the cells, as previously reported for cytokines and growth factors [61, 62].

Although most studies show that miRNAs only represent a small fraction of exosomal RNA [28, 63, 64], miRNAs transferred via exosomes can be functional in repressing their target in vitro and in vivo [23–27]. In our analysis, we found that the five most abundant miRNAs in MSC exosomes accounted for 50 % of the total miRNA reads. Thus, specific miRNAs present in high amounts might have physiological effects. In a previous study, Chen et al. [65] showed that particles secreted by human embryonic stem cell-derived MSCs are enriched in pre-miRNAs. In contrast, our study reveals that adult MSC exosomes mainly contain mature transcripts. While it is possible that the EVs released by embryonic and adult MSCs preferentially enclose different miRNA forms, conditioned medium as a whole presumably contains heterogeneous populations of RNA when compared with exosomes purified by differential ultracentrifugation, as analyzed in this study. Possibly, the pre-miRNAs are not released in association with exosomes but are incorporated into vesicles of different nature. Although there is a substantial similarity between the most represented miRNAs in ASC and BMSC exosomes, their relative proportions are different, raising the possibility that ASCs and BMSCs might deliver different information into their microenvironments. Some of these miRNAs have been implicated in MSC biology [66]. miR-486 is involved in ASC replicative senescence [67], miR-143 has been related to the immune modulatory function of MSCs [68], miR-10a and miR-22 are important regulators of MSC differentiation [69, 70], and miR-10b promotes the migration of mouse BMSCs [71]. The release of these miRNAs by ASCs and BMSCs could play a role in stem cell niche maintenance by controlling and fine-tuning proliferation, differentiation, and homing. In addition, multiple miRNAs highly represented in adult MSC exosomes regulate cell cycle progression and proliferation (miR-191, miR-222, miR-21, let-7a), and modulate angiogenesis (miR-222, miR-21, let-7f) and endothelial cell differentiation (miR-6087) [72–74]. The uptake of these miRNAs at sites of injury might promote the proliferation of multiple cell types and stimulate the formation of new blood vessels for tissue repair.

While most of the focus on functional small RNAs in exosomes has been on the class of miRNAs, in contrast with what was previously reported for immune and neuronal cells [28, 63], MSC exosomes are highly enriched in tRNAs, specifically tRNA halves, and repeats compared with the producing cells.

Recent findings suggest that tRNA pools in proliferating cells versus differentiating cells are distinct. Importantly, it was noted that genes involved in cell-autonomous functions carry codons corresponding to proliferation-associated tRNAs, while genes linked to multicellularity require differentiation-associated tRNAs [75]. Therefore it would be very interesting to investigate the correlation between the most abundant tRNA species produced and released via exosomes by MSCs and the cellular protein composition, and how this might change upon activation of specific differentiation programs.

Post-transcriptional processing of tRNAs into tRNA fragments is a nonrandom evolutionary conserved mechanism [53, 54, 76]. Strikingly, tRNA fragments of defined sizes are highly represented in adult MSCs and tRNA halves appear to be massively sorted into MSC exosomes. The 14–30-nucleotide tRNA-derived RNA fragments (tRFs) are known to associate with Argonaute proteins and have similar properties to miRNAs [53]. Moreover, these fragments can define transformed cells [77]. The function of tRNA halves in mammalian cells, however, is largely unknown. Protein biosynthesis inhibition in response to stress conditions has been initially proposed as the most plausible explanation for tRNA cleavage. However, the two halves of the tRNA are usually unequally stable, suggesting more complex roles for these tRNA pieces [54]. More recently, 5′ halves have been implicated in stress-induced translation inhibition and stress granule formation [78]. In MSCs it seems that tRNA halves are expressed at relatively high levels under standard culture conditions, possibly indicating physiological functions.

We found that ASC exosomes predominantly carry tRNA halves and are virtually devoid of full-length transcripts. BMSC exosomes have two different tRNA length profiles that seem to be related to differentiation status. Indeed, BMSCs expressing a high level of key stemness markers (BMSC II) package both full-length transcripts and 33-nucleotide fragments, while more differentiated cells (BMSC I) display the same tRNA length profile as ASC exosomes. Interestingly, however, the most abundant tRNAs in exosomes do not always correspond to those in cells, suggesting that cells can sort specific tRNAs perhaps as a mechanism for gene expression regulation. Analyzing the genomic coverage of the most abundant tRNA reads we found that ASC and BMSC I exosomes consistently display the 5′ halves of the most represented tRNA sequences, tRNA GCC (Gly) and CTC (Glu), respectively. The 5′ half of tRNA CTC (Glu) was recently shown to act in a miRNA/siRNA-like fashion to silence target mRNAs [56], although putative target transcripts for this and other fragments have not yet been identified.

The complementarity analysis of the most abundant tRNA species in cells and exosomes to the 3′ UTRs of protein-coding genes highlighted interesting putative tRNA targets involved in stem cell renewal, differentiation, and immune modulation. For instance, we found: TFCP2L1, a transcription factor involved in stem cell self-renewal [79]; RUNX2 and SOX11, master transcription factors in MSC differentiation [80, 81]; SMAD3, mediator of TGF-β-induced proliferation, differentiation, and survival; RPS19, involved in erythropoietic differentiation and proliferation [82]; and immune-related factors and inflammation mediators such as HHLA2, EMR2, and TRIM62 [83–85]. Further studies will be required to elucidate the biological function of the tRNA fragments and to evaluate whether their release via exosomes is involved in orchestrating tissue architectures.

## Conclusions

The role of EV-transferred RNA in physiological processes in vivo remains unclear, partly because the minimum amount of individual RNA molecules that is required for causing physiological changes in target cells is difficult to predict, and is likely to involve many factors. One recent hypothesis is that the most abundant and enriched RNA species in EVs play a dominant role. Using RNAseq analysis we could demonstrate that the most abundant and enriched small RNAs in adult MSC exosomes are defined tRNA species. Moreover, adipose and bone marrow MSC subtypes secrete different tRNA species that may be relevant for clinical applications. Future studies should focus on how these tRNA molecules are transported by MSC exosomes under physiological conditions and whether they influence their microenvironment in a cell type-dependent manner.

## Additional files

Additional file 1: Figure S1. Shows the Bioinformatics workflow. Figure S2 shows MSC origin (A), expression of surface markers analyzed by FACS (B), and osteogenic differentiation assessed by Alizarin red staining (C). Figure S3 shows detection of CD63 and CD81 in MSC and exosomes, PL and FBS (A), and cDNA libraries of MSC cellular and exosomal RNA (B). Figure S4 shows correlation matrix of MSC and exosome samples based on the miRNA profiles (A), relative proportion of individual miRNAs in the repertoire of total miRNA reads in cells (B), and rpm of miRNAs differentially represented in cells and exosomes (C). Figure S5 shows relative distribution of tRNAs in MSCs, LCLs, and respective exosomes (A), differentially represented tRNAs in BMSC exosomes compared with ASC exosomes (B), and length distribution of the most represented tRNAs in MSC cells and exosomes (C). Figure S6 shows tRNAs differentially represented in exosomes compared with cells. Additional file 2: Table S1. Presents antisense complementarity of the most abundant tRNA species to the 3′ UTRs of annotated protein-coding transcripts.

## Abbreviations

ALP: Alkaline phosphatase
ASC: Adipose-derived mesenchymal stem cell
BMSC: Bone marrow-derived mesenchymal stem cell
BSA: Bovine serum albumin
COL1A1: Collagen type 1 alpha 1
EEA1: Early endosome antigen A1
EM: Electron microscopy
EV: Extracellular vesicle
FACS: Fluorescence-activated cell sorting
FBS: Fetal bovine serum
FITC: Fluorescein isothiocyanate
GAPDH: Glyceraldehyde 3-phosphate dehydrogenase
LCL: Lymphoblastoid cell
α-MEM: Alpha-minimum essential medium
MSC: Mesenchymal stem cell
miRNA: microRNA
MVB: Multivesicular body
PBS: Phosphate-buffered saline
PL: Platelet lysate
rpm: Reads per million
TGF-β: Transforming growth factor beta
tRF: tRNA-derived RNA fragment
3' UTR: 3' Untranslated region
