# Supplementary material for: Human bone marrow- and adipose-mesenchymal stem cells secrete exosomes enriched in distinctive miRNA and tRNA species
Source: Stem Cell Res Ther. 2015 Jul 1;6(1):127. doi: 10.1186/s13287-015-0116-z (PMC4529699; doi:10.1186/s13287-015-0116-z)
Supplement: Additional file 1: Figure S1. — Shows the Bioinformatics workflow. Figure S2 shows MSC origin (A), expression of surface markers analyzed by FACS (B), and osteogenic differentiation assessed by Alizarin red staining (C). Figure S3 shows detection of CD63 and CD81 in MSC and exosomes, PL and FBS (A), and cDNA libraries of MSC cellular and exosomal RNA (B). Figure S4 shows correlation matrix of MSC and exosome samples based on the miRNA profiles (A), relative proportion of individual miRNAs in the repertoire of total miRNA reads in cells (B), and rpm of miRNAs differentially represented in cells and exosomes (C). Figure S5 shows relative distribution of tRNAs in MSCs, LCLs, and respective exosomes (A), differentially represented tRNAs in BMSC exosomes compared with ASC exosomes (B), and length distribution of the most represented tRNAs in MSC cells and exosomes (C). Figure S6 shows tRNAs differentially represented in exosomes compared with cells. [file 13287_2015_116_MOESM1_ESM.ppt]

## Slide 1
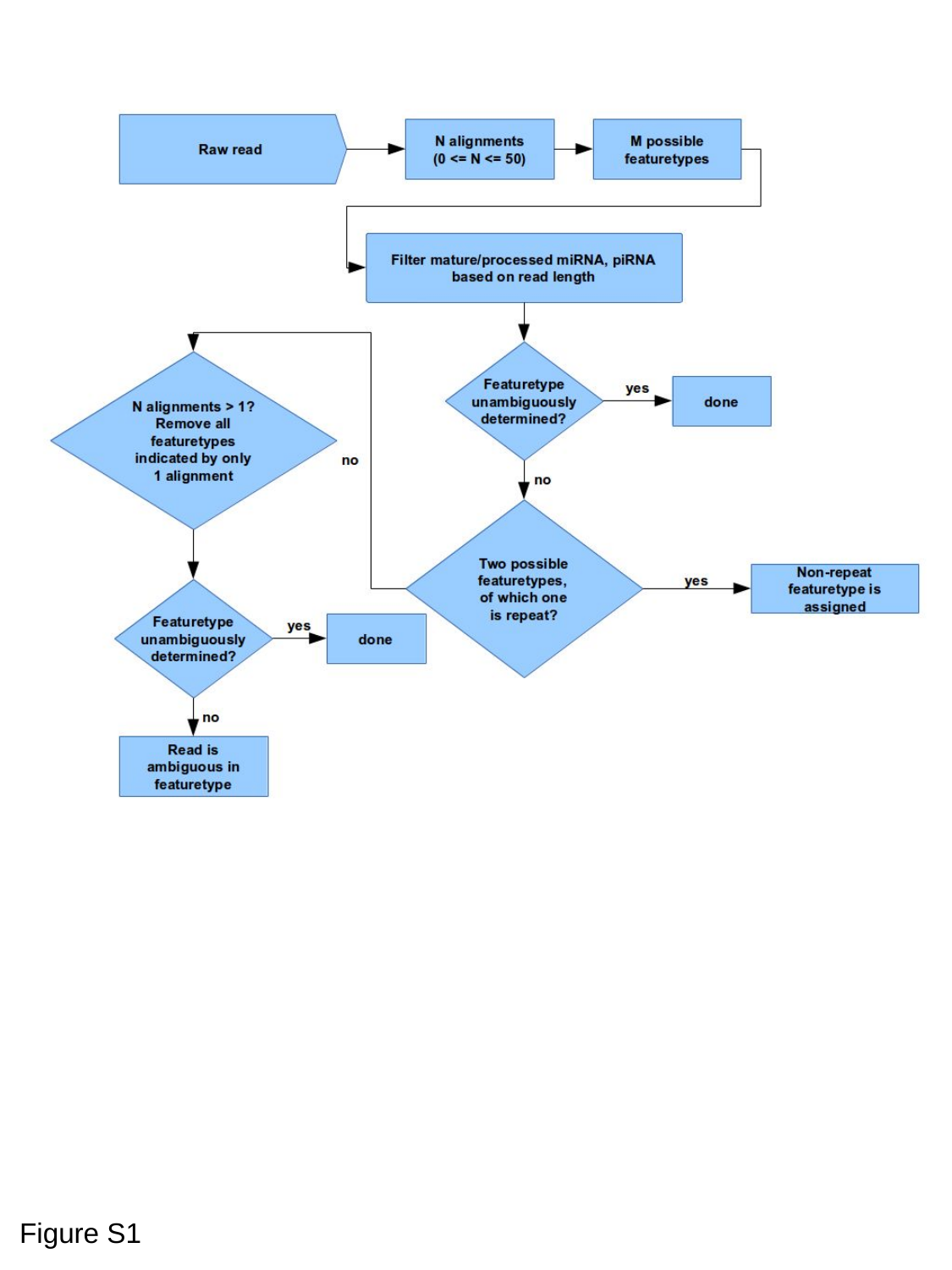

Figure S1

## Slide 2
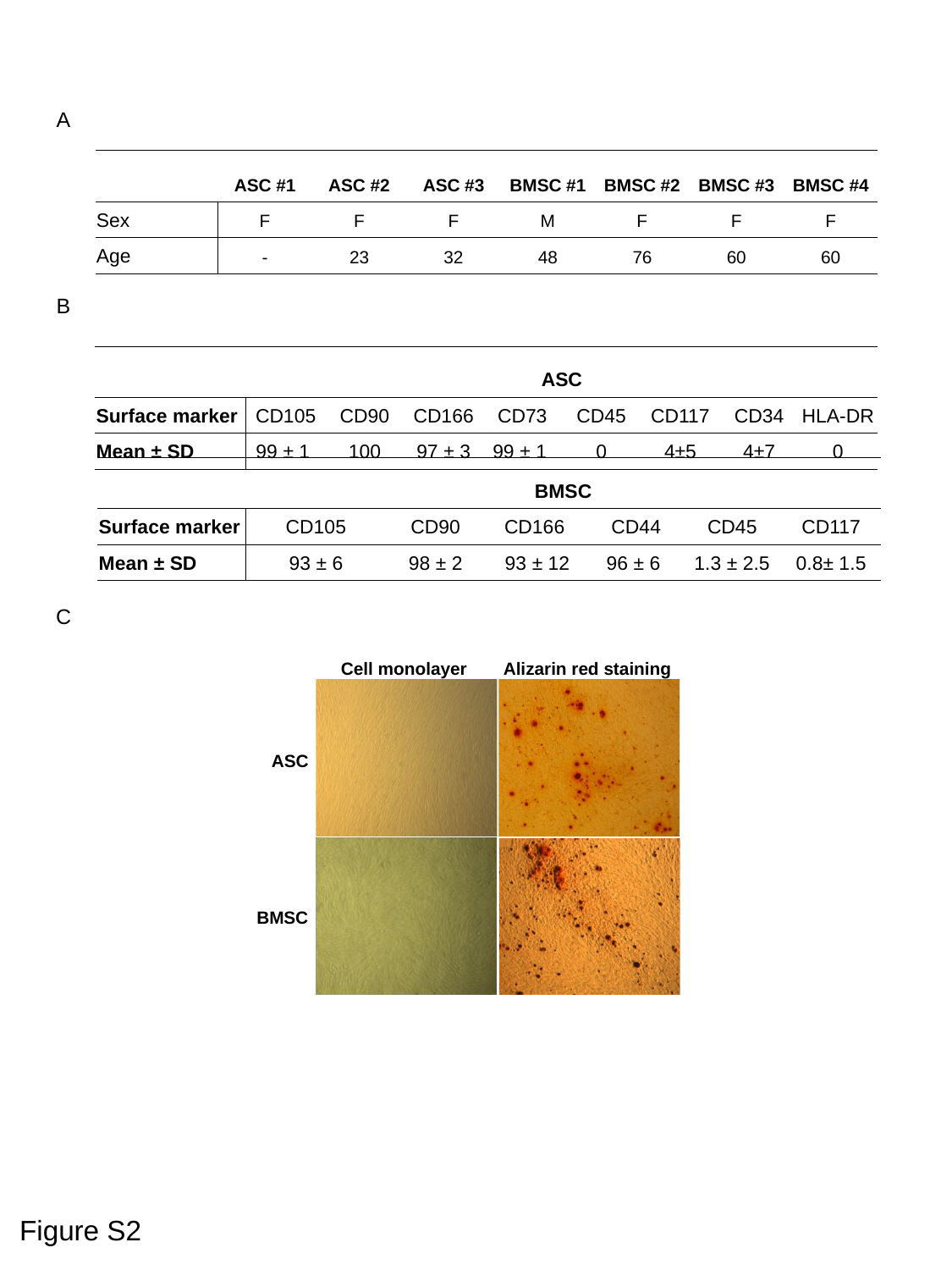

A
| | ASC #1 | ASC #2 | ASC #3 | BMSC #1 | BMSC #2 | BMSC #3 | BMSC #4 |
| --- | --- | --- | --- | --- | --- | --- | --- |
| Sex | F | F | F | M | F | F | F |
| Age | - | 23 | 32 | 48 | 76 | 60 | 60 |
B
| | ASC | | | | | | | |
| --- | --- | --- | --- | --- | --- | --- | --- | --- |
| Surface marker | CD105 | CD90 | CD166 | CD73 | CD45 | CD117 | CD34 | HLA-DR |
| Mean ± SD | 99 ± 1 | 100 | 97 ± 3 | 99 ± 1 | 0 | 4±5 | 4±7 | 0 |
| | BMSC | | | | | |
| --- | --- | --- | --- | --- | --- | --- |
| Surface marker | CD105 | CD90 | CD166 | CD44 | CD45 | CD117 |
| Mean ± SD | 93 ± 6 | 98 ± 2 | 93 ± 12 | 96 ± 6 | 1.3 ± 2.5 | 0.8± 1.5 |
C
Cell monolayer
Alizarin red staining
ASC
BMSC
Figure S2

## Slide 3
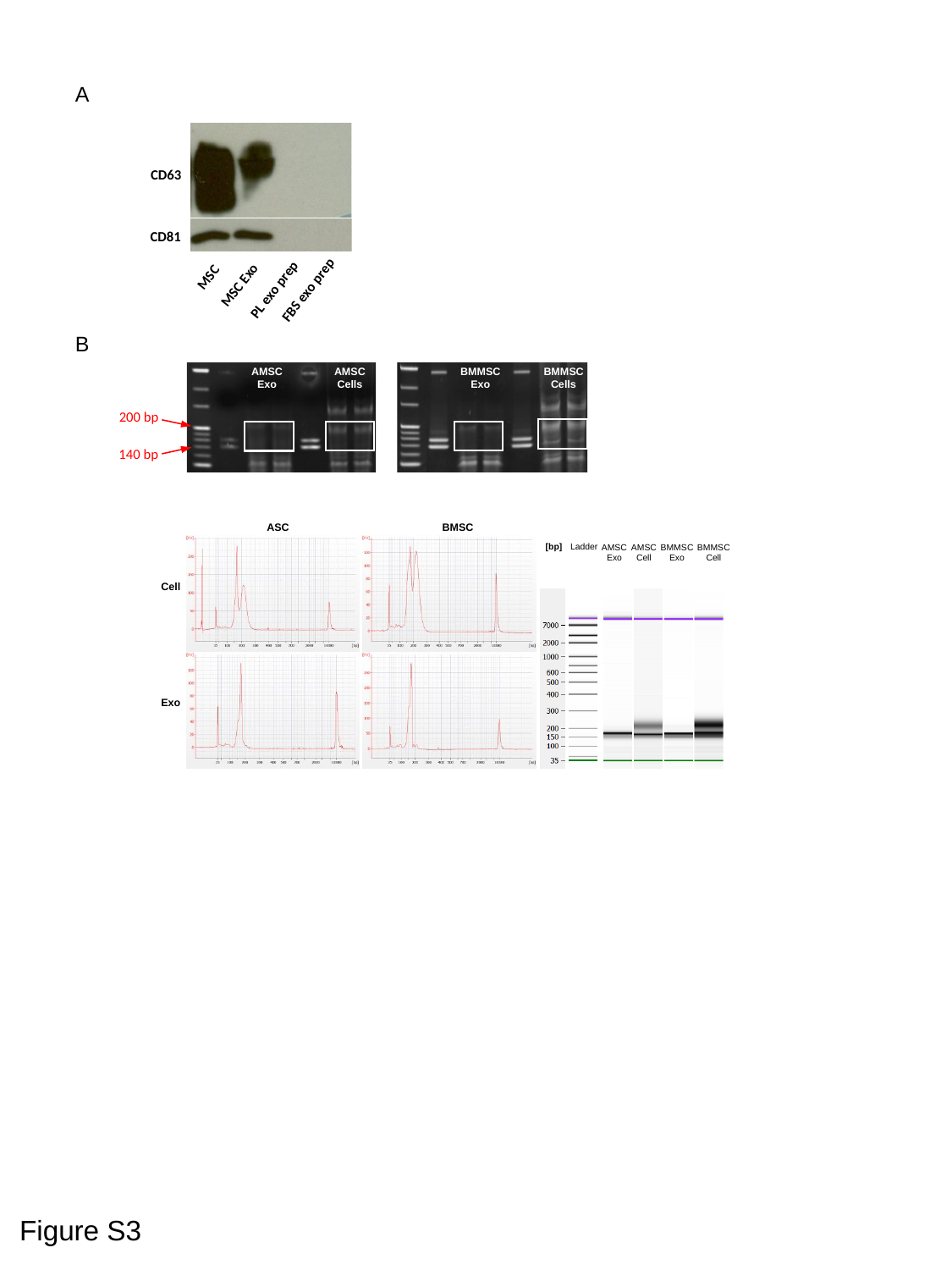

A
CD63
CD81
MSC
MSC Exo
PL exo prep
FBS exo prep
B
AMSC
Exo
AMSC
Cells
BMMSC
Exo
BMMSC
Cells
200 bp
140 bp
ASC
BMSC
[bp]
Ladder
AMSC
Exo
AMSC
Cell
BMMSC
Exo
BMMSC
Cell
Cell
Exo
Figure S3

## Slide 4
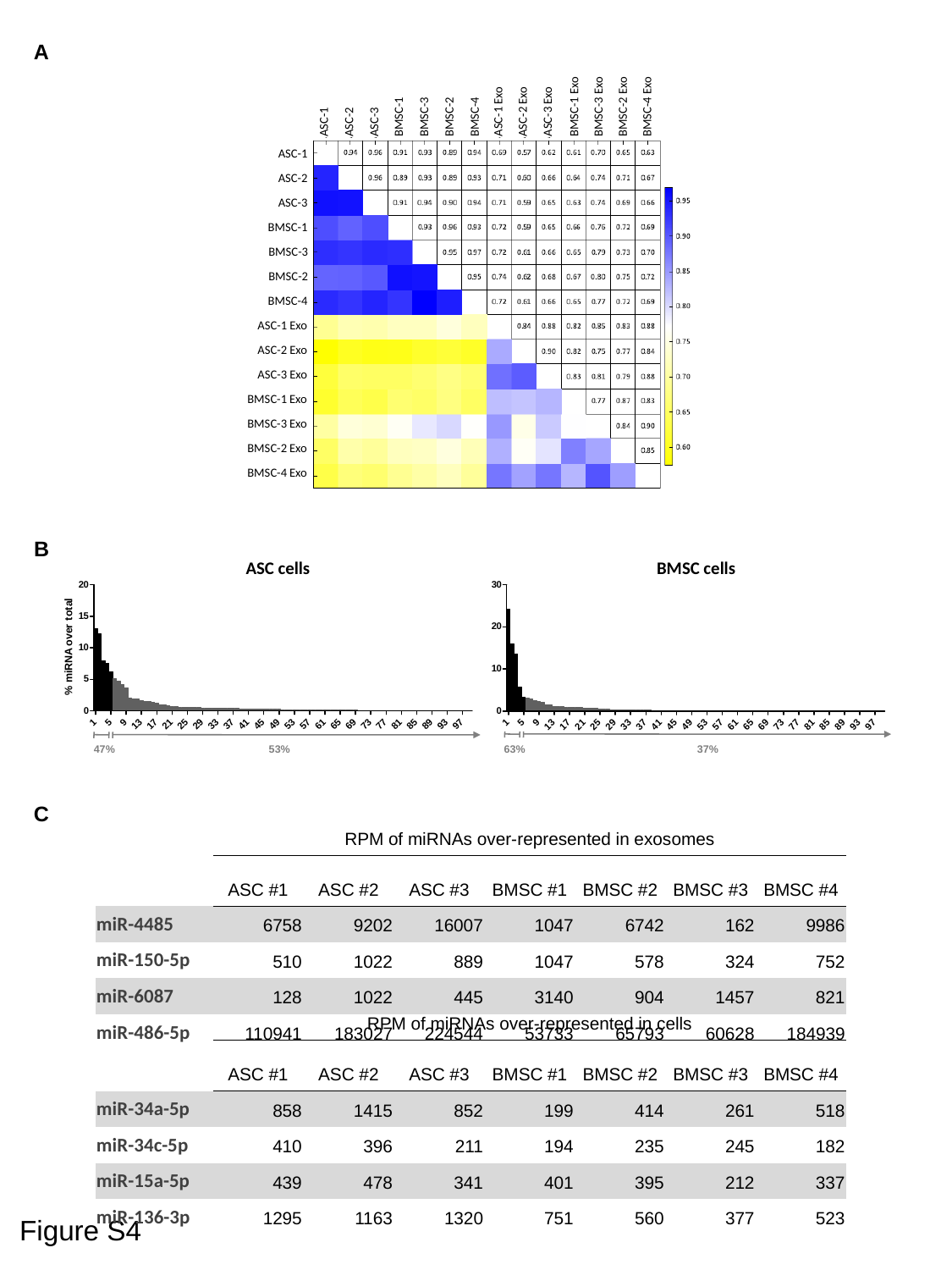

A
BMSC-1 Exo
BMSC-3 Exo
BMSC-2 Exo
BMSC-4 Exo
ASC-1 Exo
ASC-2 Exo
ASC-3 Exo
BMSC-1
BMSC-3
BMSC-2
BMSC-4
ASC-1
ASC-2
ASC-3
ASC-1
ASC-2
ASC-3
BMSC-1
BMSC-3
BMSC-2
BMSC-4
ASC-1 Exo
ASC-2 Exo
ASC-3 Exo
BMSC-1 Exo
BMSC-3 Exo
BMSC-2 Exo
BMSC-4 Exo
B
ASC cells
BMSC cells
47%
53%
63%
37%
C
| | RPM of miRNAs over-represented in exosomes | | | | | | |
| --- | --- | --- | --- | --- | --- | --- | --- |
| | ASC #1 | ASC #2 | ASC #3 | BMSC #1 | BMSC #2 | BMSC #3 | BMSC #4 |
| miR-4485 | 6758 | 9202 | 16007 | 1047 | 6742 | 162 | 9986 |
| miR-150-5p | 510 | 1022 | 889 | 1047 | 578 | 324 | 752 |
| miR-6087 | 128 | 1022 | 445 | 3140 | 904 | 1457 | 821 |
| miR-486-5p | 110941 | 183027 | 224544 | 53733 | 65793 | 60628 | 184939 |
| | RPM of miRNAs over-represented in cells | | | | | | |
| --- | --- | --- | --- | --- | --- | --- | --- |
| | ASC #1 | ASC #2 | ASC #3 | BMSC #1 | BMSC #2 | BMSC #3 | BMSC #4 |
| miR-34a-5p | 858 | 1415 | 852 | 199 | 414 | 261 | 518 |
| miR-34c-5p | 410 | 396 | 211 | 194 | 235 | 245 | 182 |
| miR-15a-5p | 439 | 478 | 341 | 401 | 395 | 212 | 337 |
| miR-136-3p | 1295 | 1163 | 1320 | 751 | 560 | 377 | 523 |
Figure S4

## Slide 5
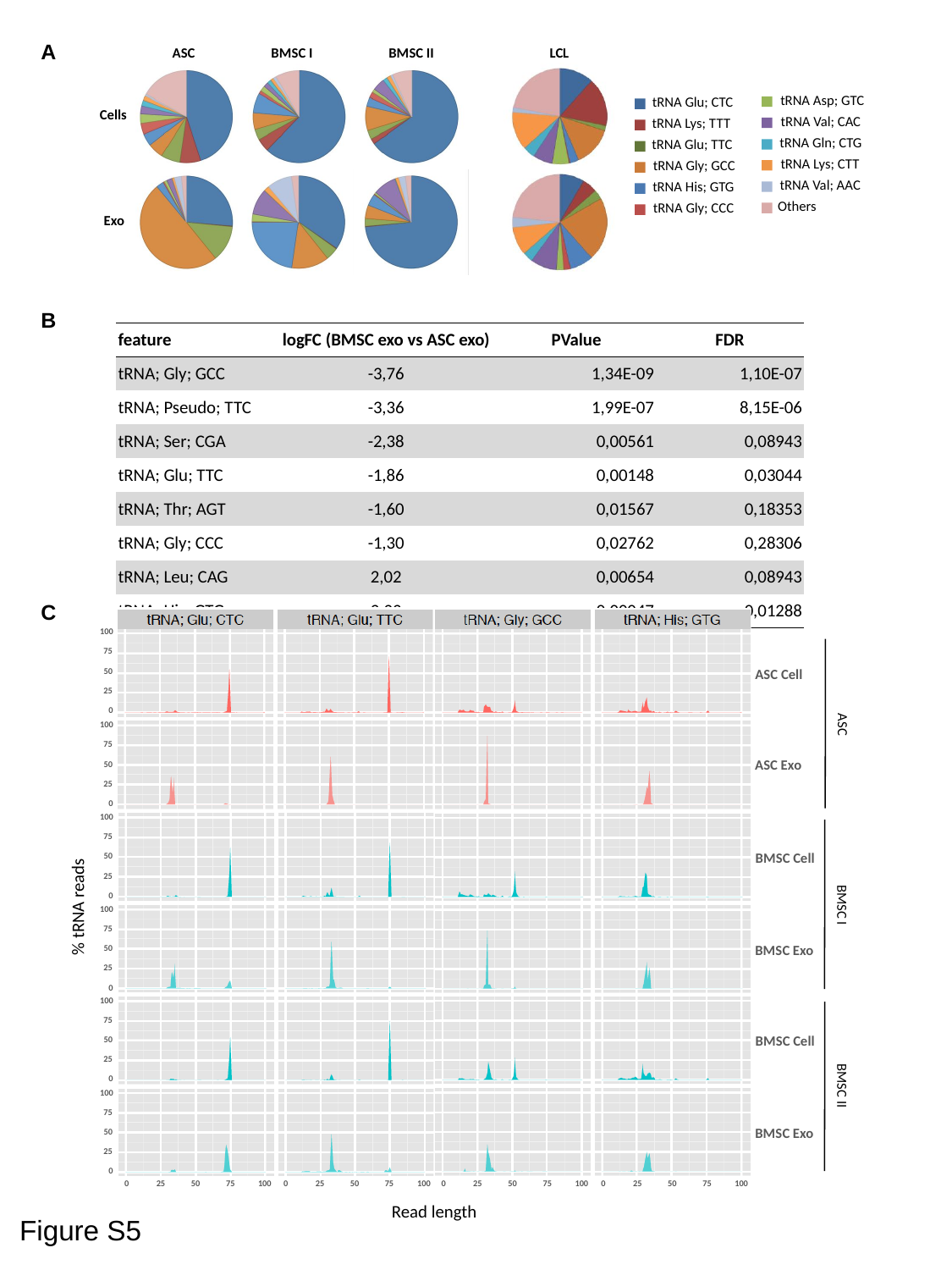

A
ASC
BMSC I
BMSC II
LCL
tRNA Asp; GTC
tRNA Val; CAC
tRNA Glu; CTC
tRNA Lys; TTT
Cells
tRNA Gln; CTG
tRNA Lys; CTT
tRNA Glu; TTC
tRNA Gly; GCC
tRNA Val; AAC
Others
tRNA His; GTG
tRNA Gly; CCC
Exo
B
| feature | logFC (BMSC exo vs ASC exo) | PValue | FDR |
| --- | --- | --- | --- |
| tRNA; Gly; GCC | -3,76 | 1,34E-09 | 1,10E-07 |
| tRNA; Pseudo; TTC | -3,36 | 1,99E-07 | 8,15E-06 |
| tRNA; Ser; CGA | -2,38 | 0,00561 | 0,08943 |
| tRNA; Glu; TTC | -1,86 | 0,00148 | 0,03044 |
| tRNA; Thr; AGT | -1,60 | 0,01567 | 0,18353 |
| tRNA; Gly; CCC | -1,30 | 0,02762 | 0,28306 |
| tRNA; Leu; CAG | 2,02 | 0,00654 | 0,08943 |
| tRNA; His; GTG | 2,22 | 0,00047 | 0,01288 |
C
100
75
ASC Cell
50
25
0
ASC
100
75
ASC Exo
50
25
0
100
75
BMSC Cell
50
25
0
% tRNA reads
BMSC I
100
75
BMSC Exo
50
25
0
100
75
BMSC Cell
50
25
0
BMSC II
100
75
BMSC Exo
50
25
0
0
25
50
75
100
0
25
50
75
100
0
25
50
75
100
0
25
50
75
100
Read length
Figure S5

## Slide 6
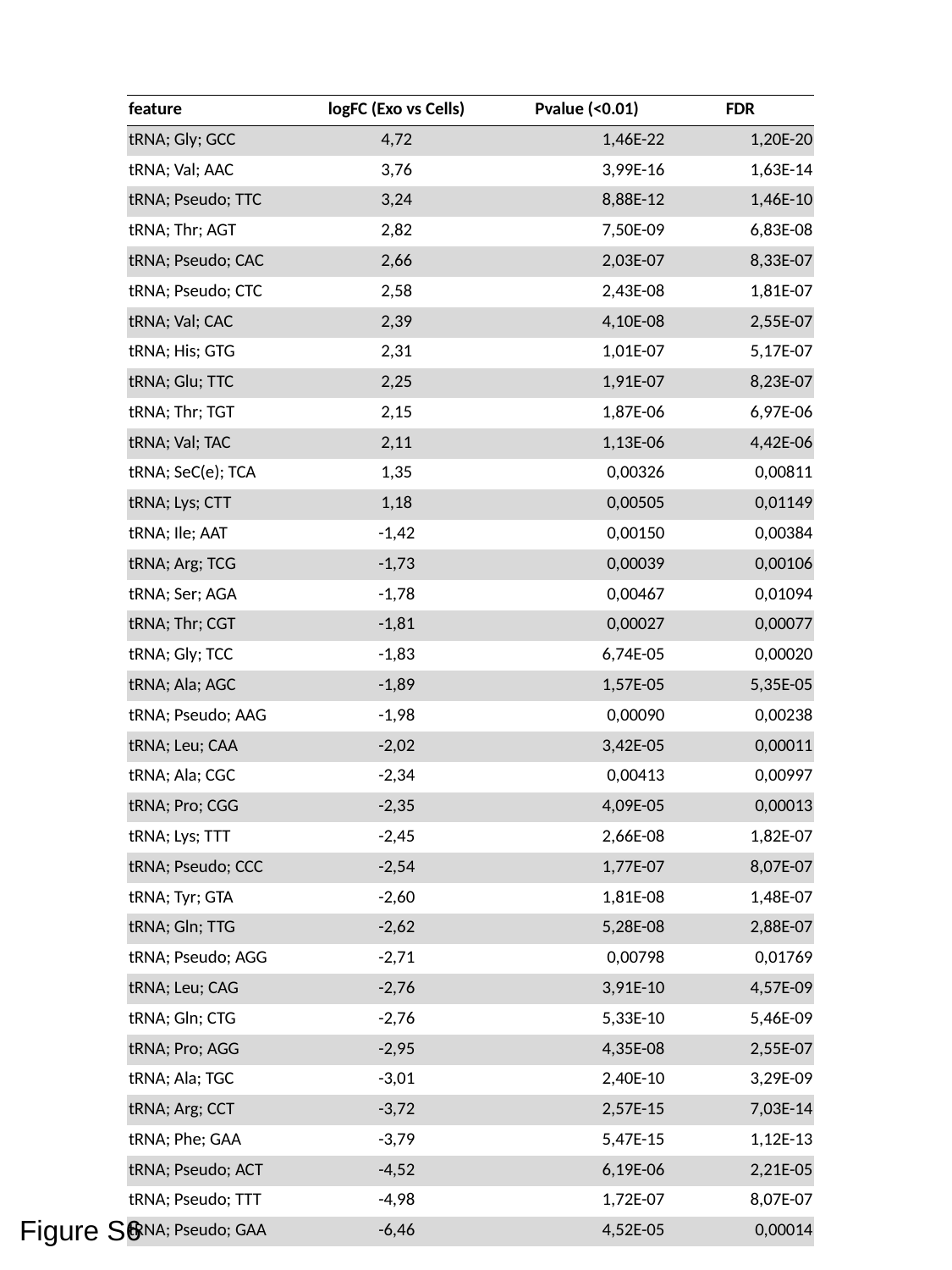

| feature | logFC (Exo vs Cells) | Pvalue (<0.01) | FDR |
| --- | --- | --- | --- |
| tRNA; Gly; GCC | 4,72 | 1,46E-22 | 1,20E-20 |
| tRNA; Val; AAC | 3,76 | 3,99E-16 | 1,63E-14 |
| tRNA; Pseudo; TTC | 3,24 | 8,88E-12 | 1,46E-10 |
| tRNA; Thr; AGT | 2,82 | 7,50E-09 | 6,83E-08 |
| tRNA; Pseudo; CAC | 2,66 | 2,03E-07 | 8,33E-07 |
| tRNA; Pseudo; CTC | 2,58 | 2,43E-08 | 1,81E-07 |
| tRNA; Val; CAC | 2,39 | 4,10E-08 | 2,55E-07 |
| tRNA; His; GTG | 2,31 | 1,01E-07 | 5,17E-07 |
| tRNA; Glu; TTC | 2,25 | 1,91E-07 | 8,23E-07 |
| tRNA; Thr; TGT | 2,15 | 1,87E-06 | 6,97E-06 |
| tRNA; Val; TAC | 2,11 | 1,13E-06 | 4,42E-06 |
| tRNA; SeC(e); TCA | 1,35 | 0,00326 | 0,00811 |
| tRNA; Lys; CTT | 1,18 | 0,00505 | 0,01149 |
| tRNA; Ile; AAT | -1,42 | 0,00150 | 0,00384 |
| tRNA; Arg; TCG | -1,73 | 0,00039 | 0,00106 |
| tRNA; Ser; AGA | -1,78 | 0,00467 | 0,01094 |
| tRNA; Thr; CGT | -1,81 | 0,00027 | 0,00077 |
| tRNA; Gly; TCC | -1,83 | 6,74E-05 | 0,00020 |
| tRNA; Ala; AGC | -1,89 | 1,57E-05 | 5,35E-05 |
| tRNA; Pseudo; AAG | -1,98 | 0,00090 | 0,00238 |
| tRNA; Leu; CAA | -2,02 | 3,42E-05 | 0,00011 |
| tRNA; Ala; CGC | -2,34 | 0,00413 | 0,00997 |
| tRNA; Pro; CGG | -2,35 | 4,09E-05 | 0,00013 |
| tRNA; Lys; TTT | -2,45 | 2,66E-08 | 1,82E-07 |
| tRNA; Pseudo; CCC | -2,54 | 1,77E-07 | 8,07E-07 |
| tRNA; Tyr; GTA | -2,60 | 1,81E-08 | 1,48E-07 |
| tRNA; Gln; TTG | -2,62 | 5,28E-08 | 2,88E-07 |
| tRNA; Pseudo; AGG | -2,71 | 0,00798 | 0,01769 |
| tRNA; Leu; CAG | -2,76 | 3,91E-10 | 4,57E-09 |
| tRNA; Gln; CTG | -2,76 | 5,33E-10 | 5,46E-09 |
| tRNA; Pro; AGG | -2,95 | 4,35E-08 | 2,55E-07 |
| tRNA; Ala; TGC | -3,01 | 2,40E-10 | 3,29E-09 |
| tRNA; Arg; CCT | -3,72 | 2,57E-15 | 7,03E-14 |
| tRNA; Phe; GAA | -3,79 | 5,47E-15 | 1,12E-13 |
| tRNA; Pseudo; ACT | -4,52 | 6,19E-06 | 2,21E-05 |
| tRNA; Pseudo; TTT | -4,98 | 1,72E-07 | 8,07E-07 |
| tRNA; Pseudo; GAA | -6,46 | 4,52E-05 | 0,00014 |
Figure S6
